# A Preliminary Study of the Efficacy of Using a Wrist-Worn Multiparameter Sensor for the Prediction of Cognitive Flow States in University-Level Students

**DOI:** 10.3390/s23083957

**Published:** 2023-04-13

**Authors:** Josephine Graft, William Romine, Brooklynn Watts, Noah Schroeder, Tawsik Jawad, Tanvi Banerjee

**Affiliations:** 1 Department of Biological Sciences, Wright State University, Dayton, OH 45435, USA; 2 Center of Life Sciences Education, The Ohio State University, Columbus, OH 43210, USA; 3 Department of Leadership Studies in Education and Organizations, Wright State University, Dayton, OH 45435, USA; 4 Department of Computer Science, Wright State University, Dayton, OH 45435, USA

**Keywords:** wearable, pre-frontal cortex, flow, flow theory, education, engagement

## Abstract

Engagement is enhanced by the ability to access the state of flow during a task, which is described as a full immersion experience. We report two studies on the efficacy of using physiological data collected from a wearable sensor for the automated prediction of flow. Study 1 took a two-level block design where activities were nested within its participants. A total of five participants were asked to complete 12 tasks that aligned with their interests while wearing the Empatica E4 sensor. This yielded 60 total tasks across the five participants. In a second study representing daily use of the device, a participant wore the device over the course of 10 unstructured activities over 2 weeks. The efficacy of the features derived from the first study were tested on these data. For the first study, a two-level fixed effects stepwise logistic regression procedure indicated that five features were significant predictors of flow. In total, two were related to skin temperature (median change with respect to the baseline and skewness of the temperature distribution) and three were related to acceleration (the acceleration skewness in the x and y directions and the kurtosis of acceleration in the y direction). Logistic regression and naïve Bayes models provided a strong classification performance (AUC > 0.7, between-participant cross-validation). For the second study, these same features yielded a satisfactory prediction of flow for the new participant wearing the device in an unstructured daily use setting (AUC > 0.7, leave-one-out cross-validation). The features related to acceleration and skin temperature appear to translate well for the tracking of flow in a daily use environment.

## 1. Introduction

Many people across the world spend time and energy on their hobbies, educational endeavors, and careers that become an everyday part of their lives. When a task’s difficulty is in balance with an individual’s level of skill, the individual becomes engaged in problem solving, which can bring a euphoric feeling, a sense of satisfaction, and other positive emotions. In order to name and coin this specific experience in research, Flow Theory was created as an explanation of these creative experiences and the motivation behind engaging in them [1]. Flow has been described and defined in many different ways, but can mainly be denoted as the process of the ultimate experience and cognitive involvement that is optimal and therefore extreme in its entirety [2]. Mihaly Csikszentmihalyi is one of the founding researchers for flow, known for studying and naming Flow Theory. According to his research, flow is experienced when the participant has clear goals and fully understands the challenge ahead. Stemming from that foundation, the participant will enter a flow state and feel intense concentration, a loss of self-awareness, and the sense that time is passing at an accelerated rate, while the task is simultaneously rewarding. Flow is a dynamic equilibrium that moves with the participant as they complete the activity. Yet, this balance can be fragile when feelings such as anxiety or boredom can turn the tide [3]. Flow tends to occur when a challenge is presented at a high enough difficulty to require creative problem solving, yet is manageable because of the level of skill needed for completing the task [4].

Increased concentration on a task that is deemed as challenging but not overwhelming is accompanied by positive behavior and reflected by physiological activation or arousal. Flow physiology is reflective of increased arousal and increased mental effort that is caused by focused attention on and effort towards a task. Flow can also be compared to stress, in that intense mental effort is demonstrated due to the high involvement and difficulty of a task [5]. Even before Csikszentmihalyi’s work in 1996, Robert Woodworth described a flow state as losing yourself in the act [6]. This is what we would commonly term today as “being in the zone”. If the skill required is higher than difficulty, the participant feels more confident in the task, which allows the likelihood of flow to increase. The threat of failure or incompletion hinders the potential for flow. Therefore, along with a balance of skill and difficulty, many environmental factors contribute to the experience of flow. The environment can spawn distractions and discouragements from maintaining a flow state [3]. The environment can also be enriching for flow, in that peaceful surroundings with minimal distraction can promote the participant to put themselves into their work fully and stay on task. Either way, the environment proves to be a factor in one’s ability to enter into and maintain a flow state.

While environmental factors may influence flow, measuring flow states can be complex. For example, qualitative surveys and anecdotal studies allow individuals to relay their experiences, but they do not necessarily reveal insights into how the brain and the body are activated during flow. Similarly, while recognizing the visual signs that someone is in a flow state is important in initially pinpointing flow, being able to understand what is happening within the human brain during flow and how this affects the body is paramount to furthering our understanding of what happens when we engage in a flow state. Measuring the physiological responses during flow that are coordinated by the brain’s specific sensory and motor regions could serve as a valuable resource in connecting what we see as the individual’s experience, what they self-report, and what physiological responses occur during flow. The prefrontal cortex is responsible in coordinating these regions together during times of higher processing for a specific task, making it an interesting source in choosing the physiological markers that would best relay the flow experience. What is needed is an understanding of how physiological indicators can be leveraged to better understand these self-reported flow states. As such, the purpose of this paper is to examine if we can use physiological indicators of engagement to predict when participants are in a self-reported flow state.

## 2. Review of Literature

Researchers have used flow as a conduit for studies that are important for understanding the cognitive experiences of students, workers, and hobbyists. Flow is important in understanding the connection between mental experiences and physical actions, and sometimes even the appearance of the person working to successfully complete a task at hand. However, as we have noted, while flow can be measured in many ways, there may be advantages to a physiological sensor that could detect these flow states. For example, in a training scenario, a wearable device could pick up physiological signals and provide haptic and cognitive feedback to an individual to help them achieve and maintain a state of flow. However, for work such as this to be possible, we must first understand what physiological signals are relevant and how to interpret them in relation to flow. As such, we begin our review of the literature with a discussion of flow, the common ways to measure flow, and some of the physiological measurements that have been considered in research thus far. We then make the case that we can likely use other, more easily captured indicators of brain activity to measure and predict these flow states. We conclude with our hypotheses about the relationships between self-reported flow states and physiological measures based on the literature.

### 2.1. Flow and Measuring Cognitive Flow

Csikszentmihalyi describes that the feeling of exhilaration caused by flow leads to an increased heart rate. He also states that a person’s body and/or mind is stretched to its limits due to the influence of flow, which leads to a display of emotion [7]. Perhaps as a result, flow and its related constructs have been measured in a wide variety of ways, ranging from self-reports to sensor-collected data. In this section, we briefly summarize some of the existing work with regard to the different types of data used.

#### 2.1.1. Eye-Tracking Data and Flow

Some studies have used eye-tracking data in relation to flow states. For example, one study used basketball and netball players’ eye movements in order to distinguish and identify these states of flow in athletes [8]. Interestingly, they found that an increased flow experience was the potential result of minimal eye movements, creating a better performance in the game for the participant [8]. Continuing with this research on eye movements, one study focused on the spatio–temporal relationship of the dynamics between eye movements and objects to analyze mental focus. Computation using eye movement scanning technology was used to study participants’ eye movements while watching videos. A linear discriminant analysis was used to compile and perceive patterns in the data. They found a correlation between increased dynamic eye movements and increased mental focus while watching a video [9]. However, eye-tracking has not been the only approach used. Researchers have also used physiological sensors to measure flow.

#### 2.1.2. Physiological Measures and Flow

The use of physiological sensors to measure cognitive flow has proven to be increasingly useful in connecting the relationship between cognitive flow to the physiological data associated with its emotional and motor influences on the body. However, research in the area is still relatively sparse.

Researchers have theorized that flow may be characterized by a decrease in the amount of task-irrelevant cognitive demands; however, research in the area has produced conflicting results [10]. It is logical, then, that some researchers have used brain-activity-related measures to examine flow. For example, in a study of electroencephalograms (EEG) and flow, researchers sought to examine if EEG-based attention measures from a less-invasive EEG machine (i.e., it used 3 dry electrodes rather than 19 wet electrodes) were related to self-reported flow. They found that there were significant correlations between the attention measure and overall flow, as well as other aspects of the flow experience [11].

Flow can also be exhibited in daily activities linked to physical responses. As such, measures of heart rate have also been of interest to flow researchers. For example, in one self-report study from university students, 15 students collected seven days’ worth of heart rate and acceleration data from a wireless ECG connected to a smartphone logging platform. The ECG values were analyzed on plots, along with heart rate variability indices. The authors found that experiences of flow were associated with an increased heart rate and an increased acceleration ratio, which correlates to sympathetic enhancement [12]. Yet, it has been proposed that the relationship between these heart-rate-related variables and flow may be individualistic and may not be linear [10].

Researchers have also shown that different types of devices can be used to collect these physiological data related to flow. For example, a project from the Palo Alto Laboratory [13] attempted to measure the affective flow states in knowledge workers by using the Kyto ear clip. An eye tracker was also used to collect the pupil dilations of both eyes. 

The data from the ear clip and subsequent surveys were combined to analyze the heart rate variability (HRV) features, skin conductivity (i.e., electrodermal activity (EDA)), and pupil diameters. The pupil diameter data proved not to be significantly useful due to the movement of the participants with respect to the screen or the lack of recorded movement of the eye; however, a strong classification performance was obtained from the other features [13].

Finally, researchers have also investigated the relationship between EDA and flow. For example, one study using video games set out to investigate the connection between flow, immersion, boredom, excitement, challenge, and fun. Using an EEG, ECG, electromyography, skin conductivity, and eye tracking, a positive correlation was discovered between the gameplay experience and the self-reported descriptions of flow [14]. Other research has found that the EDA increased as flow increased [15]. However, not all researchers have found these patterns, as one study found no significant relationship between EDA and flow [16]. Overall, it is clear that further research is needed to understand the relationship between EDA and flow [10].

As shown, various physiological measures have been examined in relation to flow. It is clear is that, in many cases, the research is relatively sparse or has produced mixed results, and the relationships between constructs may not be linear [10].

#### 2.1.3. Constructs Related to Flow and Their Physiological Measurement

As noted, flow itself can be related to other experiences such as stress or perceived difficulty. These types of outcomes have also been examined in relation to physiological data. For example, features such as heart rate variability, skin temperature, and photoplethysmograms have been used to associate work-related tasks of varying difficulties to the physical and mental state of construction workers [17]. Electrodermal activity, mean skin temperatures, HRV, interbeat intervals, and heart rate percentages were all used in a data analysis to conclude that the use of a wristband sensor can lead to the early detection of mental stressors in construction sites [17].

Sensors have also been used to measure other related constructs, such as concentration and focus. In [18], rapid eye movements, EEG alpha values, and heart rates were studied during six different cognitive tasks involving different levels of concentration. They found that a high concentration yielded many rapid eye movements, a low EEG alpha, and a high heart rate [18].

It is clear that there is no consensus on the best way(s) to measure flow, nor are there agreed upon best practices for processing these data to be able to predict flow. With this being the case, we turned our attention first to practicality: what sensors are non-invasive and commonly available? Smartwatches are very common among the general public and often include a variety of sensors. There are also research-grade wrist-worn devices that capture more data than a consumer-grade smartwatch. However, just because data exist and are capturable, this does not mean they will provide a valid metric of any given construct. Accordingly, we next turned our attention to brain physiology to understand what regions of the brain could influence and be influenced by flow, and the resulting physiological changes that may be observed.

### 2.2. The Different Areas of the Brain and Stimuli Response

To briefly summarize some relevant areas of brain physiology and their roles in cognition, behavior, and physiological responses, we draw the readers’ attention to the frontal lobe of the brain. The higher cognitive function of the frontal lobe increases cognitive flexibility. However, it is a difficult task to obtain direct measures of hypofrontality during a flow state, which allows room within the field for further investigation [19].

The frontal lobe is covered by the frontal cortex. The entirety of the frontal cortex encompasses the brain functions for influencing skeletal movement, emotion expression, speech, and visceral control. More specifically, motor reactions can be seen via the stimulation of a cortical area, including the prefrontal cortex. The orbitofrontal cortex, which is part of the frontal cortex, has shown effects within the cardiovascular system, which include blood pressure changes, heart rate, cardiac dynamics, and skin conductivity upon stimulation [20]. Many, if not all, of these indicators can be measured using non-invasive wearable sensors.

As noted, some of the indicators of flow include focused attention and effort towards a task. The ability to focus on one task and block out all outside distractions is located in the brain’s prefrontal regions. Specific neuron networks boost the efficiency of incoming signals that the participant chooses to focus on [21]. In the case of flow, our attention was drawn to the prefrontal cortex, as its main function is the execution and planning of new forms of goal-directed actions. The perception–action cycle represents the relationship between this cortex and the environment, which is also a key player in the study of flow.

#### 2.2.1. Linking Physiological Indicators to Flow Activities

Linking blood flow to certain areas of the brain, physiological data markers and flow activities are important in understanding the biological workings behind what we experience and feel in a state of flow. There are decades worth of research devoted to understanding these brain behavior relations. On the basis of prior results, neural activation through stimulation and connectivity in the medial prefrontal cortex has the ability to predict the success of an advertisement [22]. Machine learning has also been used to relate this brain activity to predictable behavior [22]. This is an important spotlight of research in the neurology field [22]. However, work also exists directly in relation to the concept of flow. For example, a study undertaken by [23] used functional near-infrared spectroscopy to examine the brain activity in the prefrontal cortex during a flow state. In their study, university students played a videogame called Tetris to induce flow and boredom. They calculated the changes in the oxygenated hemoglobin concentrations of the frontal brain regions, using the NIRS-SPM toolbox to analyze and identify the general regions of activation [23]. They found that the oxygenated hemoglobin concentration decreased during boredom and increased during flow states. A different neuropsychology study of the prefrontal cortex [24] used single photon emission computed tomography along with xenon inhalation to compare the activation of the cerebral blood flow with the performance of widely used neuropsychological tests: the continuous performance test, the Wisconsin Card Sorting Test, the Tower of London, and the Porteus Mazes. With these different tests, different regions and circuits were stimulated. The overall findings found that the activation of the regional frontal lobes occurred with cognitive challenges through the execution of the tests, except for the Porteus Mazes [24].

Blood flow and oxygen concentration also relate to a common data point for many flow studies—heart rate and heart rate variability. Higher levels of self-control exhibited and maintained by the prefrontal cortex of the brain are linked to an increase in heart rate variability [25]. More specifically, the ventromedial prefrontal cortex is a key node in decision making and evaluation. Using activity patterns from the ventromedial prefrontal cortex, heart rate variability (HRV) was associated in higher levels to these processes. HRV has been used in control and experimental conditions, meaning that it is a good form of measurement for focus. This is an important connection, because heart rate is a more accessible measure than the oxygenation of blood flow to the brain. Being able to connect physiological data with brain activity will be important in connecting experiences during tasks and their physiological effects on the human body. Using this, it will also be possible to predict what flow looks like based on the measurable physiological changes induced by the brain activity in the prefrontal region.

Finally, the physiology of the human emotional response remains poorly understood due to the ethical restrictions on the invasive experiments that would be needed to investigate. One study used photon emission to study the blood flow in the prefrontal cortex during a male orgasm. The participants were eight healthy, right-handed heterosexual males. It was found that the cerebral blood flow in all the areas of the brain, except for the right prefrontal cortex, decreased significantly [26]. Another study examined the association of emotional responses with brain activity, using joyful and prideful scenarios on various subjects in order to see how these emotions mapped out the activation across the different regions of the brain. A total of sixteen university students used a phone app to track their emotions and activities throughout the day, along with routine brain images being taken. These events usually motivated the behaviors surrounding eating, economic activities, and reproduction. The dopamine system, involved with the emotion of joy, from the ventral tegmental area all the way to the nucleus accumbens, mediates the motivation to obtain a reward from a task [27].

#### 2.2.2. Summary

The frontal cortex of the brain, including the prefrontal cortex, has been theorized to execute and perform goal-orientated actions, which include decision making, concentration, self-control, and other typical behaviors that exhibit attentiveness [25]. While this region of the brain is yet to be fully understood, it is possible to link its connection with motor and sensory functions to the theory of flow. Increased blood flow in the prefrontal regions of the brain and the selective activation of different regions based on a task’s requirements can be beneficial for the challenge of predicting the specific physiological responses as one enters a flow state. While other areas of the brain are responsible for motor and sensory skills, the prefrontal cortex is of interest because the higher thinking physiology that takes place in that area of the brain may correlate to flow. Those in a flow state are expected to exhibit higher values in the easy tasks compared to those with a higher difficulty. With that said, variability is also expected in physiological data. Variability in the heart rate, blood pressure, skin conductivity, and acceleration is expected throughout a course of activities that stimulate the prefrontal cortex.

The larger, encompassing question surrounding all the past research and investigations into the brain, sensors, and flow measurement is: what trends in physiological data are expected for those undergoing difficult and easy tasks? If there is a basic understanding of the physiological indicators of prefrontal cortex engagement, what flow is, and the balance of skill and challenge between difficult and easy tasks, then it would be feasible to connect these to the use of physiological sensors to predict flow. In its simplest form, what is understood about the prefrontal cortex’s function and motor capabilities can be directly linked to the data collected by a wearable sensor during self-reported flow states. Being able to create a predictive model for flow is extremely useful, in that students can use these models to complete their schoolwork when they are in a recognized flow state, learn more about how their brains and bodies initiate and experience higher-level thinking, and be able to continuously improve their skills, such that they continue to accomplish tasks with increasing difficulty. Predictive models are also important in the job industry when employers want to understand how engaged their workers are and if they are receiving the support they need to succeed.

## 3. Purpose of the Research

Flow is a construct of interest across many different contexts and fields, such as serious gaming [28], collaboration [29], exercise [30], education [31,32,33], and even work [34], to name a few. While flow is typically measured through subjective means, there is no widely agreed upon “best” measure [35]. As we have discussed, various physiological measures have been used as well, and the comparably limited work in the area shows that additional research is necessary. A recent review on flow concluded that the field is still working towards a comprehensive understanding of flow and how to measure it [35]. To our knowledge, there is currently no wrist-worn wearable device that provides useful automated measures of flow for students within learning contexts. Building upon previous work, we proposed a system that can accomplish this using physiological data which are readily available from a wrist-worn wearable.

Accordingly, the purpose of this study is to establish the efficacy of using wearable sensors to provide near real-time predictions of flow based on ubiquitous physiological measures. By using a wristband wearable sensor to collect the physiological data over a period of participant-chosen tasks and the prior knowledge of the prefrontal cortex’s ability to coordinate the brain regions while completing these tasks, it is possible to create a more complete picture of what is happening to an individual during a flow state. Based on prior research, the stimulation of the prefrontal cortex induces increases in EDA, heart rate variability, circulation, and body movement [25,36,37,38]. Hence, we utilize an interpretable machine learning framework to explore how physiology changes in response to the inducement of flow within the involvement and execution of tasks. We address the following questions:

(1) What types of physiological measures are most applicable to the prediction of flow?
(2) What is the efficacy of the physiological measures collected by a wearable sensor to predict an individual’s experience of a flow state during a task?
(3) How do these physiological features support a wearable device that is able to detect an individual’s flow state within an uncontrolled daily use environment?

## 4. Materials and Methods

### 4.1. Experimental Design

#### 4.1.1. Study 1: Finding the Most Important Physiological Features for Detecting Flow during Activities in a Controlled Setting

We used a 2-level block design where 60 activities were nested within five participants. Statistical power considerations indicated that 60 activities were sufficient for detecting the features with moderate effects on flow at a 95% confidence level, and the nesting of these activities within multiple participants provided a framework for discovering the features that were most useful for providing predictions which were robust across multiple device users. The participants consisted of four females and one male, all of whom were 19–20 years old and currently students at a research-intensive university in the midwestern United States. All 5 students were STEM majors, meaning that their majors were focused on the fields of science, technology, engineering, or mathematics. The participants were recruited through contacting students in the same classes or connections between other participants. The procedures were approved through the university’s Institutional Review Board (#06046).

Each participant completed two different activity sets with six different and specific tasks embedded within each of those activity sets. Since the achievement of flow requires a balance between skill and difficulty [3], the participants chose their own activities based on their personal interests or hobbies and selected tasks within each activity with a range of difficulties, in order to obtain the variance catering to their individual access to flow during the tasks. Therefore, we obtained 12 distinct tasks for each participant (Table 1), for a total of 60 tasks across the five participants. The task durations ranged between one and seventeen minutes, with an average duration of six minutes and fifteen seconds.

As seen in Table 1, each participant had twelve key distinctive data collection periods in which they had the opportunity to be in a flow state or a non-flow state. The order of the completed activities was randomized in order to eliminate the predisposed bias from the participant in the scores of the surveys or their performance during the activities. After all the activities were lined up by the tasks in a specific order, the participants wore the E4 Empatica (E4) watch during the entire duration of all the tasks. Before beginning the activities, a 5 min baseline was collected in order to obtain a participant’s baseline measurement when they were not engaged in an activity. An additional 5 min baseline was collected after the six tasks within each activity were completed. The E4 is specifically designed to combine EDA and PPG sensors to measure the sympathetic nervous system’s activity and the heart rate [39], making it a good source for data acquisition in the context of flow. After each task was completed by the participant, 9 Likert questions from the Flow State Scale created by Jackson and Marsh were completed to quantify the participant’s self-reported experience of flow during the task [40].

#### 4.1.2. Study 2: Testing the Features Derived from Study 1 for Prediction of Flow with a New Device User in an Uncontrolled Setting

Upon the selection of the most important features for the prediction of flow in Study 1, we wished to see how well these features facilitated the prediction of flow for a new participant in a comparatively uncontrolled “daily use” setting. This participant was a female freshman of 19 years of age, completing a STEM program at a research-extensive university in the midwestern United States. This participant utilized the Empatica E4 device using the same procedures as the participants in Study 1; however, this participant used the device in the context of a greater variety of activities and across a longer period of time, which is consistent with how a regular user might use the device. When the participant noticed her degree of flow changing during an activity, she documented her state of flow on the Flow State Scale [40] and then started a new session. Otherwise, the nine questions from the Flow State Scale [40] were completed at the end of each activity. This participant collected data in the context of 10 sessions across a 2-week time span. The activities included journaling (1 session), academic writing (1 session), exercising at the gym (2 sessions), lifting weights (1 session), cartoon drawing (2 sessions), and playing Zelda on a Gameboy video game system (3 sessions). The task durations ranged between 10 and 83 min.

### 4.2. Instrumentation

#### 4.2.1. Use of the Empatica E4

To predict the students’ cognitive flow based on physiological parameters, the E4 wearable device collected 6 different measures from each data session. The E4 has four sensors, including a photoplethysmography sensor, an EDA sensor, a 3-axis accelerometer, and an optical thermometer [39]. The measures utilized in this study included: EDA (µS), skin temperature (TEMP) (°C), heart rate (HR) (beats per minute), and acceleration (ACC) on three orthogonal axes (X, Y, Z). The E4 wristband collected the heart rate at a frequency of 1 Hz, the skin temperature and EDA at 4 Hz, and the acceleration at 32 Hz [39]. False readings were removed at the start of each session as the Empatica E4 device calibrated and stabilized. The first 20 s of data were removed from all the readings. The EDA, temperature, and acceleration were also edited to remove false readings by integrating their sampling frequencies of 4 Hz (EDA and temperature) and 32 Hz (acceleration). Therefore, the first 80 readings of the EDA and temperature were removed, along with the first 640 readings for the acceleration. The initial 10 readings for the heart rate were removed, which was sampled at 1 Hz. It is important to note that the heart rate was calculated as a moving average of the previous 10 s of data; hence, the first 10 s of readings were removed. In Study 1, with 60 tasks executed by 5 participants, over 10 h of data (over 36,000 heart rate measures, 144,000 skin temperature and EDA measures, and 1,152,000 acceleration measures) were collected to be used for further analysis. In Study 2, in the context of 10 tasks undertaken by a single participant over a 2-week period, over 7.5 additional hours of data (over 27,000 heart rate measures, 108,000 skin temperature and EDA measures, and 867,000 acceleration measures) were collected in order to test the efficacy of the features derived from Study 1 within a comparatively uncontrolled daily use context.

#### 4.2.2. Modeling the Measurement of the Flow State Scale

After each task was completed by the participant, a survey was also completed corresponding to each individual task, in order to get a measure of the flow during that task. All the questions were the same for each survey, along with the same answer scale that included the answer options: “strongly agree, agree, neutral, disagree, or strongly disagree”. These questions were derived from the Flow State Scale in order to maintain questions that were solely focused on the participant’s experience with flow [40]. The original 36-item instrument, which was tested on 394 athletes aged 14–50 in the context of performing athletic activities in their chosen sport, was structured as a 9-dimensional assessment that contained 4 parallel questions, aligning with each of the 9 elements of flow: (1) the challenge–skill balance, (2) action–awareness merging, (3) clear goals, (4) unambiguous feedback, (5) the concentration on the task at hand, (6) a sense of control, (7) a loss of self-consciousness, (8) the transformation of time, and (9) the autoletic experience. In the interest of a shorter survey and given our interest in obtaining a single measure for flow instead of nine distinct measures, we selected one question per element of flow in order to construct a nine-item survey which we initially hypothesized to be unidimensional.

One potential problem with this approach is that a unidimensional model was shown to provide an inadequate representation of the data using a confirmatory factor analytic (CFA) approach [40]. Specifically, each of the 9 elements of flow was moderately correlated (r = 0.224–0.795), leading the authors to conclude that the 9 elements of flow may be related through a higher-order factor; this was supported through their CFA approach [40]. In short, there is little consensus from the data on how to best use the Flow State Scale in a parsimonious way and leaves a considerable gray area as to how the Flow State Scale should be used to measure flow in this study. For example, the authors note that the presence of a higher-order factor does not necessarily imply that flow can be treated as a single score, but given that the nine dimensions are correlated with each other, a 9-dimensional model likely has an unnecessary level of complexity [40].

To inform us of how we should use the Flow State Scale in this study, we needed to obtain a more detailed understanding of how it worked as a measure of flow for our participants. To this end, we used a Rasch modeling approach, using the responses from Study 1 [41]. The Rasch approach to exploring the validity of the constructs relies on providing a fixed definition of the measurements based on the law of measurement invariance and evaluating the validity of the instrument based on its conformity to this law. The information about the instrument’s functioning is gathered through an inspection of the anomalies in the data with respect to the model, which is held to be true a priori, which aligns well with the post-positivist hypothetico-deductive approach to inquiry and the process of falsification [42]. This can be contrasted with the confirmationist approach used by Jackson and Marsh [40], where increasingly complex models were fitted to the data until an acceptable level of fit was achieved.

We used the Rasch Rating Scale Model [43] as a definition of measurement, which allowed us to evaluate the efficacy of the ideal case, where the nine elements of flow are used to construct a single measure for flow. With this model, the rating scale was fixed across all the nine elements of flow, and the probability of a participant attaining a particular level for an element of flow was defined to be proportional only to the difference between the participant’s level of flow and the difficulty of achieving that particular element of flow. In order to inform our decision on how to use this scale to yield a measure for flow, we focused on three questions: (1) to what extent do the nine elements of flow conform to the unidimensional hierarchy defined by the Rasch Rating Scale Model [43], (2) are there particular elements of flow that do not fit well into the flow construct, and (3) what does a full flow experience look like? The conformity to the unidimensionality assumption was evaluated using a principal components analysis (PCA) on the residuals with respect to the Rating Scale Model. Random residuals, indicated by a first eigenvalue of less than 2, indicated a conformity of the nine elements of flow to the unidimensionality assumption, implying that the nine elements of flow can be treated as a unidimensional hierarchy. If a first eigenvalue greater than 2 was observed, then the loading of the particular elements of flow onto the residual factors was inspected in order to tease out which elements of flow fell outside of the main factor of interest [44]. An observation of the elements of flow that did not fit well was carried out through analysis of misfit of the response pattern for each element of flow with the model-expected response pattern. Mean squares infit (information-weighted) and outfit (outlier-sensitive) indices were used as the measures of fit [45]. These have expected values of 1 but can range between 0.5–1.5 for productive measures [46]. Values above 1.5 (worse-than-expected fit) are of the greatest concern, as these would indicate that a particular element of flow is biased in favor of the participants with lower flow states, which contradicts the intended directionality of the scale [45]. Values below 0.5 (better-than-expected fit), although less of a concern, indicate that an element of flow as it is defined contains other dimensions which artificially bias it in favor of those who tend toward higher flow states [47].

### 4.3. Calculation of Distribution Features from the Physiological Signals

A total of 30 distributional features were calculated from the physiological measures for each task. For the heart rate, skin temperature, EDA and acceleration in the x, y, and z directions, we calculated the following features for each activity within each individual: a mean change with respect to the baseline, a median change with respect to the baseline, a change in the standard deviation with respect to the baseline, skewness of the measures during the task, and kurtosis of the measures during the task. The purpose of the mean and median changes with respect to the baseline was to quantify the average change in the value of the measure due to the task. The change in the standard deviation with respect to the baseline was calculated to provide insight into how the variability of the parameter changed due to the engagement in the task. The skewness was calculated for each physiological parameter in order to quantify the directionality of the outlying data, where a positive skewness indicated extreme values in the positive direction and a negative skewness indicated extreme values in the negative direction. Finally, the kurtosis was calculated for each physiological parameter in order to quantify the peakedness/tail thinness of the distribution. A high kurtosis indicated smaller tails, indicating the tendency for the measures to take more similar values. A lower kurtosis, which is indicative of larger tails, indicated a greater tendency toward multimodality.

### 4.4. Inferential Analysis of the Relationship between Physiology and Flow

A 2-level stepwise fixed effects logistic regression model with SPSS 21 was utilized as an interpretable framework for selecting the features which served as significant predictors of the outcome of achieving flow. With this approach, the “participant” was first forced into the model as a factor. After controlling for the unique effects of the individual participants, we used a forward stepwise model building procedure [48] in order to select the features that improved the fit of the model after accounting for the participant-level differences [48]. We used a 90% confidence level based on the likelihood ratio test as a criterion for the addition of a feature to the model. One limitation of stepwise regression is that, as a greedy approach, it does not necessarily identify the best set of variables [49]. We therefore used a more liberal Type 1 error rate so that the features providing robust predictions across the participants were added to the model, with the understanding that the potential for overfitting would be assessed using a between-participant cross-validation framework [50] that evaluated the efficacy of the features in providing predictions that were robust across the individuals.

### 4.5. Using Machine Learning to Evaluate Efficacy for Prediction of Flow

We utilized two interpretable machine learning algorithms, logistic regression and naïve Bayes, to explore the efficacy of the retained features in predicting whether or not participants were experiencing a flow state. Logistic regression is a discriminative classifier which directly models the probability of being in a flow state conditioning on the feature inputs. A naïve Bayes is a generative classifier which learns the joint probability of whether or not a participant is in a flow state and uses that participant’s feature measures as inputs [51]. Predictions are then made using the Bayes rule to calculate the probability of being in a flow state given the physiological measures. Given that we had 60 tasks which were given flow state labels based on the Flow State Scale [40], it was instructive to utilize both generative and discriminative classifiers, given the research suggesting that, while discriminative classifiers such as a logistic regression have a lower asymptotic error, generative classifiers like naïve Bayes tend to approach their asymptotic error quicker, which increases their efficacy when less training data are available [52].

In order to obtain an idea of the efficacy of physiological data for predicting whether or not a participant is in a flow state, we used three model training–testing scenarios. First, we used participant-fold cross-validation, where we trained the algorithm on the data from four participants and tested it on the fifth participant. This was repeated for each respective participant. This style of validation was of the greatest interest in this study, in that it delivered the most direct insight into how well a flow detection device that utilizes the statistically significant physiological features would perform on a new individual. Second, we used participant-stratified 10-fold cross-validation, which allowed the algorithms to make predictions about a participant’s flow state after seeing some of the other data from the same participant, as well as from the other participants. Third, we tested the algorithms on the same data that were used for training, which provided a best-case scenario for the prediction. Furthermore, the difference in the predictive efficacy between these different cross-validation methods is indicative of the extent to which the models were overfitting.

In sum, person-fold cross-validation indicates how well a flow detection device would work right away on a new participant, while 10-fold cross-validation indicates how the prediction might improve if a participant went through a short training phase where they manually labeled whether or not they were in a flow state. Since no model is perfect, we must negotiate a trade-off between Type 1 (false positive) and Type 2 (false negative) errors. For this, we evaluated the efficacy of each algorithm and cross-validation strategy by using precision and recall. Precision refers to the ability of the model to identify only the flow states. A low precision means we will detect many non-flow states (higher Type 1 error). Recall refers to the ability of the model to detect the observed flow states. A low recall means we will miss many actual flow states (higher Type 2 error). We used F1 score and the area under the ROC curve as omnibus measures for the performance. The F1 score was calculated as the harmonic mean of the precision and recall. The ROC curve was constructed as a graph of the true positive rate vs. the false positive rate for a range of probability cutoffs, and the area under the curve (AUC) was used as a measure of the algorithm performance [51]. All the machine learning was implemented using the sci-kit learn package in Python.

## 5. Results

### 5.1. Analysis of the Flow State Scale

The Rasch Rating Scale Model with the Study 1 data indicated that the nine elements of flow provide a hierarchy that exhibits some departure from unidimensionality. The eigenvalue of 2.76 on the first residual factor was greater than the cutoff of 2 suggested in the simulation work to indicate a reasonable conformity with the unidimensionality assumption [44]. The infit indices for the individual elements of flow ranged between 0.62 and 1.43, and the outfit indices ranged between 0.68 and 1.60. Loss of self-consciousness was the only element of flow that fell above the upper bound of 1.5 that was recommended by [46]. Given that it also had the highest loading onto the residual dimension (l = 0.79), we can conclude that this particular element of flow is discordant with the other elements. The other elements with high loadings included the challenge–skill balance (l = 0.64) and unambiguous feedback (l = 0.54). In this sense, although the nine elements of flow make up a reliable scale (reliability = 0.86), the elements are not necessarily unidimensional.

**Figure 1:**
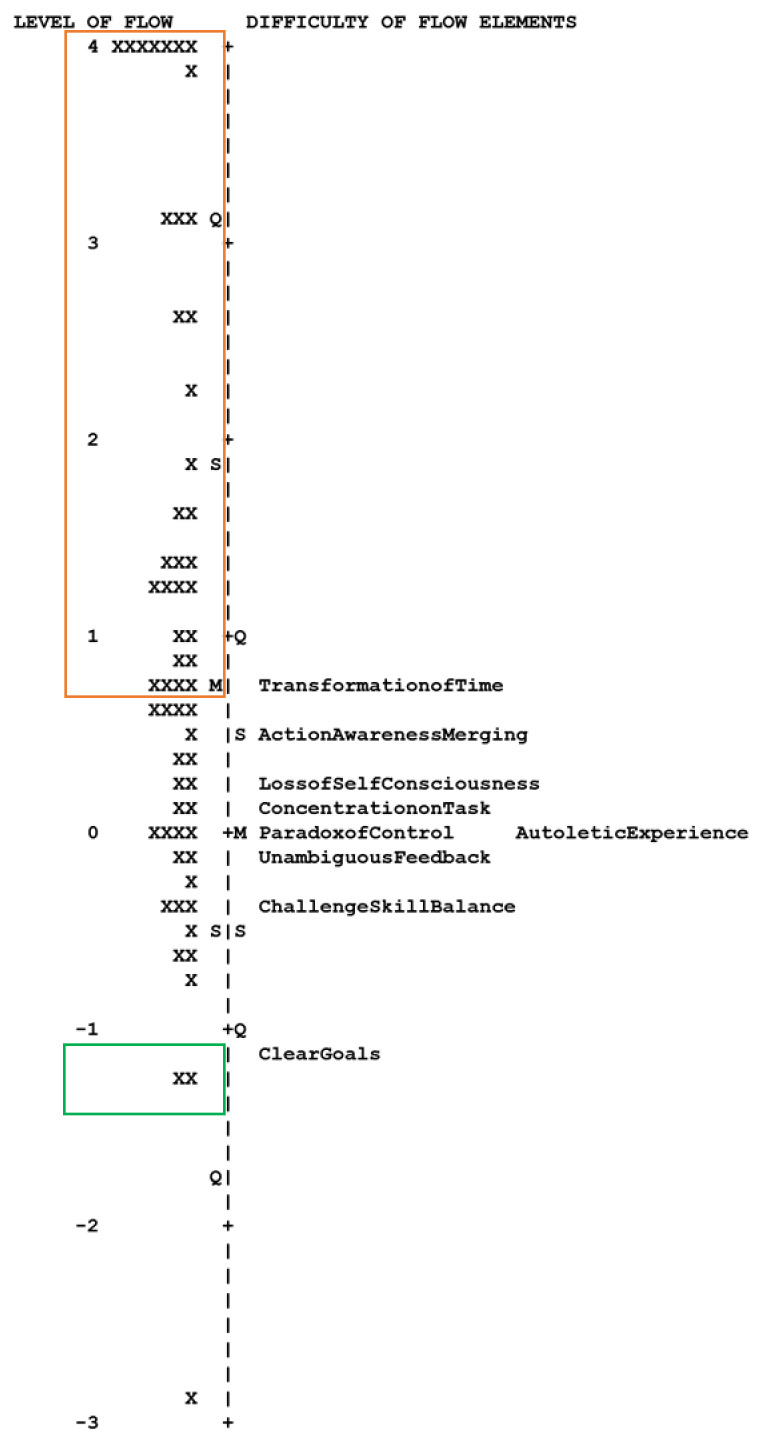
A map of the level of flow achieved during a task and the difficulty level of the nine flow elements on the Rasch logit scale. The green box indicates activities by participants achieving no flow and the orange box indicates activities that achieved full flow according to the Rasch Model. Activities between the boxes indicate partial flow.

With this in mind, it is useful to look at what a full flow experience might look like based on the hierarchy of the elements of flow, in terms of their difficulty in achievement. In the map below (Figure 1), the level of flow measured during each activity (reliability = 0.86) and the difficulty of each element of flow (reliability = 0.91) are plotted on a common log-odds scale derived from the Rasch Rating Scale Model. The difficulty levels for the elements of flow represent the center of the rating scale, which is the point at which a participant is equally likely to select the highest rating (strongly agree) and the lowest rating (strongly disagree). It follows that a measure above the difficulty level for an element of flow implies a higher likelihood of a high rating than a low rating for that element of flow. Conversely, a measure below the difficulty level implies a higher likelihood of a low rating than a high rating. Using this representation, we observe that the different elements of flow are experienced sequentially. As a person’s immersion in a flow state increases, they will first experience a clear vision of their goals. To progress further, they will need to report a balance between their skill and the challenge of a task. After this, the person will experience a sense of control and reward. A progressively higher immersion in flow is accompanied by a loss of self-consciousness and action–awareness merging. The transformation of time is the last element of flow to occur, and its position at the top of the Rasch hierarchy suggests that it will only occur after the other elements of flow have occurred. We highlight this in Figure 1, which shows the activities where the participants did not experience flow at all (green box) and the ones where they reported a full flow experience (orange box). The activities between the boxes indicate a partial flow experience, which included some of the easier elements, but not the more difficult elements. In this sense, we decided to define flow in this study as the reporting of a full flow experience, which is indicated by a participant’s reporting of “agree” or “strongly agree” for the transformation of time. For the subsequent analyses, a report of “agree” or “strongly agree” for the transformation of time was coded as a 1 (a full flow state achieved), and all the other responses were coded as a 0 (a full flow state not achieved).

### 5.2. Feature Selection and Explanation

When the five participants were forced into the model for the achievement of a full flow experience, the model was significant at the 95% confidence level (χ^2^ = 11.59, df = 4, *p* = 0.021), indicating some individual differences in how flow was reported. We found no significant relationship between the frequency of the reported flow states and the temporal sequencing of the tasks within each activity (r = 0.16, *p* = 0.77, r^2^_adjusted_ = 0), and so our models treated the sequencing of the tasks as arbitrary. After attempting to add the 30 features to this model using the forward stepwise algorithm, 5 features were selected: (1) the change in the median skin temperature with respect to the baseline (likelihood ratio χ^2^ = 4.78, *p* = 0.029), (2) the skewness of the skin temperature measures (likelihood ratio χ^2^ = 10.57, *p* = 0.01) (3) the skewness of the acceleration in the x direction (likelihood ratio χ^2^ = 9.14, *p* = 0.02), (4)the skewness of the acceleration in the y direction (likelihood ratio χ^2^ = 12.64, *p* < 0.001), and (5) the kurtosis of the acceleration in the y direction (likelihood ratio χ^2^ = 14.63, *p* < 0.001). Adding these variables improved the model’s fit significantly compared to the model with just the participants (χ^2^ = 29.45, df = 5, *p* < 0.001) and resulted in a statistically significant final model (χ^2^ = 41.04, df = 9, *p* < 0.001).

Based on these five variables (Table 2), the data indicate that skin temperature and acceleration are the key physiological features to look at when attempting to draw robust inferences on the experience of flow from a wearable sensor. The finding that we found most interesting was the relationship between flow and the increase in skin temperature with respect to the baseline (OR = 10.3, *p* = 0.063). This implies that a flow experience is accompanied by vasodilation, which allows for circulation to the extremities. This is backed up by the fact that a more negative skewness in the skin temperature was associated with being in a flow state (OR = 0.0756, *p* = 0.0077). A negative skewness occurs when the bulk of the skin temperature values shift in the positive direction and the values left at the lower tail of the distribution pull the mean in the negative direction. This likely explains why the difference in the median skin temperature, as opposed to the difference in the mean skin temperature, ended up being a significant predictor.

With regard to acceleration, the higher-order distributional features (the skewness and kurtosis) ended up providing robust predictions, as opposed the lower-order features (mean and variance). Flow was associated with a more positive skewness for the acceleration in the x direction (OR = 5.24, *p* = 0.017) and a more negative skewness for the acceleration in the y direction (OR = 0.205, *p* = 0.011). Given that the differences in the magnitude (mean or median) with respect to the baseline were not significant, this meant that there was enough non-systematic variance in the participants’ movement during the activities to render the difference non-detectable. However, the skewness statistics show that the bulk of the values for the acceleration in the x direction shifted to lower values during a flow state, while those for the acceleration in the y direction shifted to higher values. The negative relationship between the kurtosis for the acceleration in the y direction and the flow (OR = 0.741, *p* = 0.0072) indicates that, during a flow state, the acceleration in the y direction became more platykurtic, meaning that the tails of the distribution got heavier. This implies a heavier presence of the values further way from the center of the distribution. In sum, these relationships imply that a flow state is accompanied by a change in the activity level and an associated activation of the sympathetic vasodilator system [53], which would explain the increase in the skin temperature with respect to the baseline and a shift in the distribution toward higher skin temperature values.

### 5.3. Efficacy for Prediction of Flow

#### 5.3.1. Study 1: Between-Participant Efficacy in a Controlled Setting

Using the five statistically significant features from Table 2, both logistic regression and naïve Bayes provided models for achievement of flow that were robust to the differences between the participants (Table 3 and Table 4). The logistic regression performed best, with an AUC of 0.77 and an F1 measure of 0.72 (confusion matrix in Table 4). As expected, the classification performance improved when the algorithm was tested on the data used for training (resubstitution) (AUC = 0.86, F1 = 0.80). However, this degree of improvement was modest. In sum, our data indicate that these five features comprise a classifier for flow that is robust across different users and is resistant to overfitting. Hence, this classifier shows promise as a framework for a wrist-worn wearable that is capable of detecting flow.

#### 5.3.2. Study 2: Transfer to an Uncontrolled Daily Use Setting

The use of the specific coefficients in Table 2 performed poorly for the prediction of the new participant’s report of flow during the 10 activities (AUC = 0.50), which was not surprising given the significant individual-level variability shown in Study 1. However, when the model was re-trained using the same features and tested using leave-one-out cross-validation, the predictive efficacy of the flow detection sensor was similar to that which was found within the between-participant cross-validation framework in Table 3 (AUC = 0.71, F1 = 0.70 for logistic regression; AUC = 0.71, and F1 = 0.80 for naïve Bayes) (Table 5).

**Figure 2:**
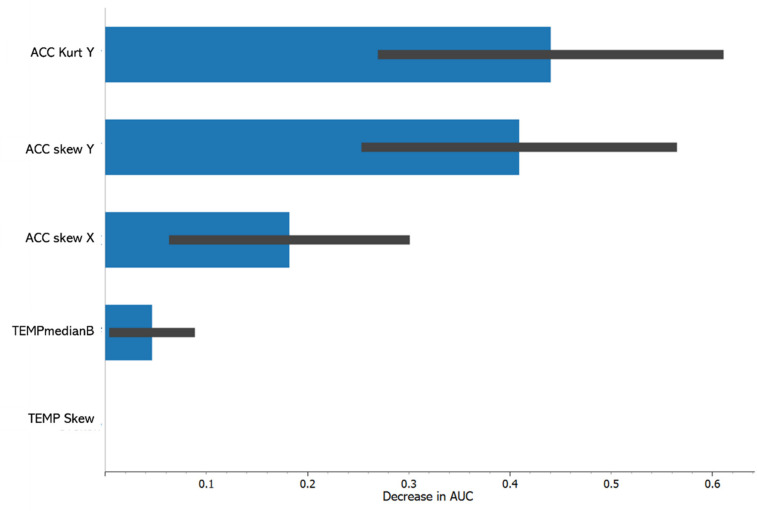
Relative importance of the five features in terms of the average decrease in the AUC value for the Study 2 logistic regression model associated with randomly shuffling a feature in the data. 1000 random shuffles were used for each feature. Mean (blue bar) and standard deviation (black line) for reduction in AUC across the 1000 random shuffles is shown.

**Figure 3:**
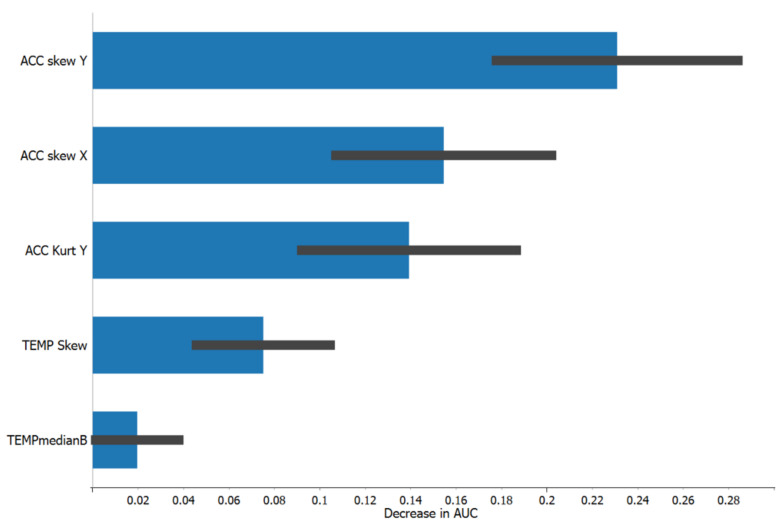
Relative importance of the five features in terms of the average decrease in the AUC value for the Study 1 logistic regression model associated with randomly shuffling a feature in the data. 1000 random shuffles were used for each feature. Mean (blue bar) and standard deviation (black line) for reduction in AUC across the 1000 random shuffles is shown.

Given the satisfactory performance of the features for the prediction of flow in both studies under rigorous cross-validation procedures, which emphasize between-user generalizability, we were able to explore and compare the relative importance of the individual features by using the permutation method in the Python sci-kit learn package. With this method, the data within one feature were randomly shuffled and their effect on the predictive efficacy (a reduction in the AUC value) was found. This was performed for 1000 permutations for each feature, and the means (blue bar) and standard deviations (black line) for the subsequent reductions in the model’s AUCs are shown (Figure 2 and Figure 3). This analysis shows that not only are the five features transferrable to a new participant, but their relative importance to the prediction model bears a lot of similarity. Although there were some differences in the order of importance between Study 2 (Figure 2) and Study 1 (Figure 3), the acceleration features (the kurtosis and skewness of the acceleration in the Y direction and the skewness of the acceleration in the X direction) were the most important, while the temperature features (the skewness of the temperature and the difference in the median temperature from the baseline) were less important for the prediction of flow in both study contexts.

## 6. Discussion

The state of flow is described as an optimal state of productivity which occupies a middle ground in the continuum between boredom and anxiety [7]. An optimal state of flow requires a balance between parasympathetic and sympathetic brain activity [54]; in this sense, it is not surprising that multiple studies have identified a relationship between flow and sympathetic enhancement [12,55]. As the prefrontal cortex is involved in the cognitive control of both movement and sympathetic enhancement due to the regions it coordinates, the effects of flow, such as blood circulation (skin temperature), electrodermal activity, acceleration, and heart rate, should be related to the prefrontal cortex’s activity. Measuring these specific data markers can be useful in relating the stimuli and motor activity that are needed to experience flow to how we can understand and quantitatively pinpoint a flow state in an individual.

Our data point to a promising new methodology for using the easily obtainable physiological data from wearable sensors to better understand and detect flow. While cognitive flow is experienced by the individual as a mental process involving the prefrontal cortex, the data indicate that flow is accompanied by certain measurable physiological manifestations. In designing a sensor to detect flow, our data indicate that the distributions of skin temperature and acceleration are the most important features to utilize. An upward shift in skin temperature measurements while doing a task is indicative of vasodilation due to the arousal of the sympathetic nervous system [53], which may result from the activation of the anterior cingulate/dorsolateral prefrontal cortices. Indeed, Csikszentmihalyi described flow in terms of a feeling of exhilaration, noting that when a person’s body and/or mind is stretched during a flow state, the person will experience an increased level of stress and tend toward a display of emotion [7].

The importance of skin temperature makes a case that vasodilation may indicate an immersion in flow, whereas vasoconstriction may indicate being out of flow. It is difficult, however, to explain systematically where acceleration, and in particular acceleration along certain axes, fits into flow. We believe that there is a connection between movement and the state of flow [38]. Physical activities, specifically those of moderate to high intensities, have been known to correlate positively with mental engagement [56]. However, why would flow be associated with a positive shift in the skewness for the acceleration in the x direction but a negative shift in that for the acceleration in the y direction? Additionally, why is flow associated with a lower kurtosis of the distribution for the acceleration in the y direction? Based on the previous work conceptualizing flow [7], it is reasonable to state that the flow experience necessitates engagement in a single repetitive activity for some duration of time, and so it follows that movement will be associated with flow in many cases [38,56]. However, it is currently unclear whether movement has a causal connection to flow—for example, a change in movement behavior is associated with the increased state of stress [57] that is associated with flow—or whether it is connected to flow through the actual performance of a particular task [56]. In summary, we do not believe that a lack of movement necessarily indicates a lack of mental engagement. However, it is no surprise that movement is an effective measure of the mental flow state when used in conjunction with the other physiological measures within a machine learning framework. To this end, our use of a between-participant cross-validation framework takes into account the systematic differences in the movement between the participants and the unique tasks in which they were engaging—acceleration artifacts that are not related to the state of flow would be expected to hinder the classification performance within this framework. Our strong classification performance suggests that movement was useful for predicting the flow of a new participant, outside of the participants that were used to train the model, which is the type of generalizability we want to see in a wearable device designed to detect the flow in a new user. Although teasing out the causal connection between movement and flow may not be immediately essential for the development of an effective wrist-worn sensor, it may help to make acceleration a more useful feature for the detection of flow in future developments.

Our discussion so far has focused on the five significant features. However, we found it surprising that the features related to the heart rate and EDA were non-significant, given that these are also associated with the sympathetic responses in the brain. For example, given the established relationship between heart rate variability and the activation of the prefrontal cortex [25], we expected to find a significant increase in both the magnitudes and standard deviations of the participants’ heart rates with respect to the baseline when they entered a flow state. Furthermore, increases in EDA are associated with sympathetic nervous system arousal [58] and the activation of the anterior cingulate/dorsolateral prefrontal cortices [36], and so we were surprised that a change in the EDA from the baseline was not a significant predictor for flow. Although changes in the distributions of the heart rate and EDA are associated with prefrontal cortex activity and sympathetic neurological processes in general, we believe these were more difficult to detect within our study design, which focused on generalization across multiple participants. Specifically, both heart rate and EDA varied greatly from one individual to another, which made their use across participants challenging. By comparison, body temperature is relatively constant from one individual to the next, which may make a change in skin temperature a phenomenon that is more repeatable across multiple participants, making it easier to detect vasodilation due to the sympathetic neurological activation that is associated with flow.

## 7. Limitations and Future Research

Based on our results, we were able to establish connections between the physiological responses that are indicative of the current state of flow. Given that the models created were imperfect, there are certain measures that can be taken in order to improve these models for future research. Our findings provide evidence for both internal and external validity; however, a larger sample of tasks from a single participant or multiple participants is a logical next step. Our research focused on a sample of college students pursuing studies in science, technology, engineering, or mathematics (STEM), which delivers generalizability to that specific context. In future studies, generalizability outside of the context of collegiate studies would require a more diverse sample. The goal is for the results found in studies such as this to be applicable to the general public. Hopefully, as flow research continues into using broader and more extensive samples, our ability to discover and utilize smaller effects will be improved. Indeed, more data would also allow for inferential models such as logistic regression to learn the data trends with a higher confidence, which would reduce the Type 2 error rates and allow for the detection of relationships with smaller effect sizes. More diverse data which stem from a larger pool of participants and a larger variance in activities would decrease the context-dependency of the models, but may also lessen the context-specific interpretability due to the larger variance in the activities [59].

In addition, measures from other wearable sensors may be useful in collecting and identifying flow. The current analysis establishes the importance of changes in the distributions of acceleration and skin temperature in the detection of flow. However, one notable weakness of the current study is that we were not able to detect meaningful relationships between the distributions of heart rate, EDA, and flow, despite the literature linking the activity in the prefrontal cortex with changes in both the heart rate variability [25] and EDA [36]. It is possible that these indicators vary based on the activity being performed for other reasons. For example, if a participant is in a flow state in a first-person simulation video game, their heart rate might be accelerated and speed up or slow down depending on the specific situation in the game. Both heart rate and EDA would also be increased by repetitive exercise through increased oxygen demand and sweating. On the other hand, if that participant is working through a more homogeneous activity such as a Sudoku puzzle, their heart rate may not vary as much, even if they are fully in a flow state. It makes sense then that the features related to heart rate and EDA may vary considerably across both activities and individuals. To the end of addressing hypotheses like these in future research, it is useful to note that some wearable sensors, such as the Hexoskin vest, have been specifically designed for the collection of cardiorespiratory data such as heart rate, heart rate variability, breathing rate, and minute ventilation, in addition to data on activity and movement [60]. Since breathing rate and volume are also associated with the sympathetic response [61] and may be more repeatable from one participant to the next than heart rate and EDA, adding a sensor for respiratory monitoring may improve our ability to predict flow, even if the sample sizes remain relatively small. Furthermore, since breathing rate and heart rate are correlated, data on breathing rates might help researchers to better parametrize the variability of heart rate across individuals.

Finally, we were surprised that HRV did not come up as a significant predictor of flow in our models, given that HRV is correlated with pre-frontal cortex activity [25], which is related to flow [23]. The measure of HRV that was used in this study (the standard deviation of the heart rate during the activity) is a time domain measure which bears a strong mathematical similarity to the HRV measures reported in other neurology research, such as the standard deviation of the RR interval (which is used to calculate the heart rate in bpm) and NN interval [25]. Time domain measures such as this are suitable for time durations between 1 min and 24 h, which encompass the activity durations in this study; however, researchers have also acknowledged that differences in the activity length can affect the accuracy of the HRV measures [62]. Although an exhaustive analysis of these HRV features is beyond the scope of the current study, we acknowledge that the use of a single HRV measure is a limitation of this study, as there are many features we could have used from the time domain, frequency domain, and non-linear measures [62]. More research is needed to understand the uniqueness and redundancy of these different features, in terms of the information they provide and the effect of the activity length on the accuracy of these measures. Given the relationship between the HRV and pre-frontal cortex activity [25], which is also related to flow [23], we recommend an empirical study which explores, in detail, how the different HRV measures relate to each other, as well as the unique information they can provide that may help us to understand more holistically the usefulness of HRV in the prediction of flow.

In summary, the E4 data in the context of our study design is sufficient for obtaining useful predictions for flow. However, we are optimistic that our ability to detect flow will only improve as the number of training data increase and sensors that focus on the measurements of cardiorespiratory features are incorporated. With this in mind, we propose the creation of a wearable system or device that detects flow and gives the user feedback in near real-time. This would first involve the collection of data using a device such as the E4 or Hexoskin sensor. Using a simple machine learning algorithm, such as a logistic regression or naïve Bayes, a prediction could be made within the smartphone app itself, without requiring an export to a cloud service. Once a prediction is made, the device could either silently record or send an alert that the user has achieved flow, and the user’s flow record could be stored for future reference. Our data indicate that reasonable predictions can be achieved using models developed on the data from other individuals. However, the app could also invite the user to respond to the Flow State Scale periodically, in order to better train the model for the individual.

## 8. Conclusions

In this preliminary study that draws on data from a small sample of participants, we identify physiological measures which are useful for predicting flow and evaluate the efficacy of a wrist-worn wearable system for measuring flow. Our data indicate that an upward shift in skin temperature and changes in acceleration are the strongest indicators for flow, which is likely due to the relative constancy of the baselines of these indicators across individuals. Additional data, and perhaps additional sensors, are needed in order to better understand the role and efficacy of heart rate and EDA for predicting flow. In any case, both the logistic regression and the naïve Bayes algorithm illustrate promise for the efficacy of predicting flow in near real-time by using physiological data, which sets the stage for a wearable flow detection device which provides the user with feedback through a smartphone app. To this end, it is useful to consider that it is preferable to have a system that is specific as opposed to sensitive. In other words, a system that misses some existing flow states is preferable to a system that gives false alarms. For example, a ROC analysis of the between-person cross-validation indicated that, if the probability threshold for the logistic regression algorithm was set to 0.304, then a true positive rate of 55% and a true negative rate of 82% could be achieved, meaning that this system would detect over half of the flow experiences, but would correctly identify four out of the five non-flow experiences. For now, we advocate for a device that focuses on a high specificity and are hopeful that its sensitivity will continue to improve as more and different types of data become available. As these conclusions were derived from a small sample of university students, we would like to see our models tested on additional samples and further validated with data from other sensor systems. In addition, it would be interesting to evaluate the efficacy of our findings for predicting flow in other levels of students, as well as in working participants engaging in activities related to their careers or hobbies. We hope that the creation of a novel wrist-worn wearable for detecting flow will not only move research on engagement forward, but will also inspire the lay public to improve their ability to focus on and fully engage in tasks that enrich their lives.

## Figures and Tables

**Table 1:** Summary of activities and embedded tasks completed by the participants.

	Activity 1			Activity 2		
	**Playing the Violin**			**Solving Sudoku Puzzles**		
**Participant 1**	The Scientist (Coldplay)	La Cinquantaine (Vivaldi)	Surprise Symphony (Haydn)	Easy puzzle	Easy puzzle	Easy puzzle
	18 (Josephine Trott)	Concerto in G Major (Vivaldi)	The Giga (Corelli)	Hard puzzle	Hard puzzle	Hard puzzle
	**Playing Video Games**			**Reading**		
**Participant 2**	Minecraft	Mario Kart	Nintendo Dogs	Hide & Seek	Spillover	The Gone World
	Super Mario 64	Luigi’s Mansion	Super Smash Bros	Zone One	American War	Biophysical Journal Article
	**Playing Video Games**			**Reading**		
**Participant 3**	MLB 2021	Assassin’s Creed	Call of Duty (Easy Mode)	Sports Research Article	Hide and Seek	Oh the Places You’ll Go!
	Call of Duty (Hard Mode)	Madden (Hard)	UFC	Fluid dynamics textbook	Economy Learning Handbook	Astrophysics book
	**Drawing**			**Reading**		
**Participant 4**	Draw simple shapes	Draw a picture of a hill covered with flowers	Draw a picture of favorite pet	Goodnight Moon	The Giving Tree	The Very Hungry Caterpillar
	Draw a picture of a family of birds in a nest with eyes closed	Draw an entire detailed forest landscape in 5 min	Draw a sea monster with eyes closed	Scientific Journal	Scientific journal	Scientific journal
	**Playing Video Games**			**Drawing**		
**Participant 5**	Apex Legends (normal)	God of War	Animal Crossing	Free-hand draw for 10 min	Use simple shapes to create a monster	Draw a picture of Rowdy Raider using a reference pic
	Outlast	Apex Legends (Ranked)	Super Smash Bros	Draw detailed ocean scene in 5 min	Draw a self-portrait with eyes closed	Draw 10 cats while talking about personal opinion on pollution

**Table 2:** Statistically significant features for prediction of flow after controlling for between-participant differences (not shown).

Feature	Estimate	SE	Wald χ^2^ (df = 1)	*p*-Value	Odds Ratio	Explanation
Difference in Median Skin Temperature	2.33	1.26	3.46	0.063	10.3	Flow is associated with an increase in skin temperature
Skewness of Skin Temperature	−2.58	0.969	7.10	0.0077	0.0756	Flow is associated with more negative skewness in skin temperature
Skewness of Acceleration in the X Direction	1.66	0.695	5.67	0.017	5.24	Flow is associated with more positively skewed acceleration in the x direction
Skewness of Acceleration in the Y Direction	−1.59	0.624	6.47	0.011	0.205	Flow is associated with more negatively skewed acceleration in the y direction
Kurtosis of Acceleration in the Y Direction	−0.300	0.112	7.23	0.0072	0.741	Flow is associated with a more platykurtic distribution of acceleration in the y direction

**Table 3:** Performance of logistic regression and naïve Bayes algorithms for prediction of flow.

	Between-Participant Cross-Validation				Stratified 10-Fold Cross-Validation				Resubstitution			
Algorithm	AUC ^a^	F1 ^b^	Precision	Recall	AUC ^a^	F1 ^b^	Precision	Recall	AUC ^a^	F1 ^b^	Precision	Recall
Logistic Regression	0.77	0.72	0.75	0.72	0.82	0.74	0.75	0.73	0.86	0.80	0.81	0.80
Naïve Bayes	0.70	0.64	0.66	0.63	0.68	0.62	0.62	0.62	0.85	0.80	0.81	0.80

^a^ Area under the receiver operator characteristic curve; and ^b^ harmonic mean of precision and recall.

**Table 4:** Confusion matrices for the logistic regression and naïve Bayes algorithms using between-participant cross-validation.

	Logistic Regression Prediction		Naïve Bayes Prediction	
Observed	No Flow	Flow	No Flow	Flow
No Flow	26	12	24	14
Flow	5	17	8	14

**Table 5:** Confusion matrices for the logistic regression and naïve Bayes algorithms using the features derived from Study 1 for prediction of flow in a new participant.

	Logistic Regression Prediction		Naïve Bayes Prediction	
Observed	No Flow	Flow	No Flow	Flow
No Flow	3	1	3	1
Flow	2	4	1	5

## Data Availability

Data are available upon request by contacting the corresponding author. Data are not publicly available due to privacy restrictions.
